# Phenolic Compounds from *Hibiscus sabdariffa* L.: Extraction, Microencapsulation and Application in a Buttermilk-Based Fermented Dairy Beverage

**DOI:** 10.3390/foods15203628

**Published:** 2026-10-12

**Authors:** Andressa Ladeira Bernardes, Marcella Duarte Villas Mishima, Jhonatan Faria da Costa, Sidney Rodrigues de Jesus Silva, Herlândia Cotrim Santos, Evandro Martins, Jane Sélia dos Reis Coimbra, Renê Chagas da Silva, Izabela Maria Montezano de Carvalho, Ana Clarissa dos Santos Pires, Maria do Carmo Gouveia Peluzio

**Affiliations:** 1 Department of Nutrition and Health, Universidade Federal de Viçosa, Viçosa CEP 36570-900, Minas Gerais, Brazilmpeluzio@ufv.br (M.d.C.G.P.); 2 Department of Food Technology, Universidade Federal de Viçosa, Viçosa CEP 36570-900, Minas Gerais, Brazil; 3 Department of Physics, Universidade Federal de Viçosa, Viçosa CEP 36570-900, Minas Gerais, Brazil; rene.silva@ufv.br

**Keywords:** bioactive compounds, anthocyanins, antioxidants, buttermilk, functional dairy product

## Abstract

Phenolic compound extracts from *Hibiscus sabdariffa* L. (HS) exhibit promising functional and technological properties; however, their application in foods is limited by their susceptibility to degradation, making microencapsulation a promising strategy. This study aimed to test different extraction conditions for phenolic compounds from HS, microencapsulate the HS extract, incorporate the selected microcapsules into a fermented dairy beverage based on buttermilk (a dairy co-product) containing *Lactobacillus gasseri* (*L. gasseri*), and evaluate stability during 28 days of refrigerated storage. The use of 70% cereal alcohol resulted in the highest recovery of bioactive compounds and antioxidant capacity. The microcapsules exhibited an acidic pH, solubility exceeding 75%, encapsulation efficiency ranging from 48.86% to 92.12%, and heterogeneous morphology across samples. The beverage demonstrated satisfactory physicochemical stability and slow degradation of phenolic compounds (half-life [t½] of 34.5 days). Finally, ultra-high-performance liquid chromatography coupled with tandem mass spectrometry (UPLC-MS/MS) confirmed the presence of 14 compounds in the HS calyces, microcapsules, and beverage, indicating the retention of these compounds through to the final product. Overall, microencapsulation enabled the incorporation of bioactive HS compounds into a fermented dairy matrix, confirming their potential as a functional ingredient and natural colorant.

## 1. Introduction

The growing consumer demand for natural, functional, and sustainable foods has driven the replacement of synthetic additives with plant-derived bioactive ingredients capable of providing health benefits while simultaneously performing technological functions in food products. Among these compounds, phenolic compounds stand out due to their high antioxidant capacity and potential anti-inflammatory, antimicrobial, and cardioprotective effects, being widely associated with the reduction in chronic disease risk [1,2,3,4]. In this context, *Hibiscus sabdariffa* L. (HS), commonly known as hibiscus, represents a promising food and medicinal plant, traditionally consumed in the form of infusions, beverages, and culinary preparations. Its calyces are also widely used in the preparation of teas and acidic-tasting beverages, due to their characteristic color and flavor. HS contains high concentrations of anthocyanins, flavonoids, and phenolic acids [5,6]. In addition to its biological activities, the anthocyanins present in HS are responsible for its characteristic reddish-purple coloration, making this plant a promising functional ingredient and natural dye for food applications [6,7,8]. Therefore, its traditional food use, combined with its rich composition of bioactive compounds, justifies the choice of HS as a promising plant matrix for the recovery and application of phenolic compounds.

Despite this potential, the technological application of HS phenolic compounds still faces important limitations. These compounds are susceptible to degradation during food processing and storage, particularly due to exposure to oxygen, light, temperature, and pH variations, which may compromise their stability, antioxidant activity, bioavailability, and coloring capacity [9]. Therefore, the development of effective strategies to protect these compounds is essential for expanding their application in food products.

Microencapsulation has emerged as an effective strategy for improving the stability, solubility, bioavailability, and shelf life of bioactive compounds [10]. This technology involves entrapping active compounds within a wall material, forming particles that protect them from adverse environmental conditions while potentially enabling controlled release [9,11]. Among the most widely used techniques, spray drying and freeze-drying are particularly relevant because of their broad applicability, although they differ in processing conditions, retention efficiency, and the structural characteristics of the resulting particles. In addition to the drying method, the choice of encapsulating agent plays a decisive role in the efficiency and physicochemical properties of the microcapsules [9,12].

Different wall materials exhibit distinct physicochemical properties and can significantly influence the protection and retention of bioactive compounds. Maltodextrin is widely used due to its low cost, high solubility, low viscosity, and good film-forming ability [13]. Inulin, besides acting as an encapsulating agent, exhibits prebiotic properties, potentially increasing the functional value of the final product [14]. Whey protein concentrate (WPC), in turn, possesses excellent emulsifying properties and can form protective matrices that enhance the retention of bioactive compounds during processing [15,16,17]. Although these wall materials have been widely investigated for the microencapsulation of phenolic compounds, the effects of their combinations and proportions in association with different drying methods on the retention and stability of HS phenolic compounds remain insufficiently understood.

The improved stability provided by microencapsulation expands the possibilities for incorporating these compounds into different food matrices [18]. Among them, fermented dairy beverages stand out due to their consumer acceptance, nutritional value, and potential as carriers of bioactive ingredients and probiotic microorganisms [19,20]. Furthermore, microencapsulated phenolic compounds can simultaneously act as natural antioxidants, functional ingredients, and natural colorants, contributing to the development of value-added food products [21,22,23].

Buttermilk represents a particularly interesting dairy matrix for this purpose. As a by-product of butter production, buttermilk contains milk fat globule membrane components, including proteins, phospholipids, and glycolipids, which exhibit relevant technological and functional properties [24,25]. Its incorporation into fermented beverages represents a sustainable strategy for valorizing this dairy by-product, contributing to circular economy approaches and the development of foods with enhanced nutritional and functional value [26]. Furthermore, the incorporation of probiotic microorganisms may further enhance the functional potential of these beverages. Among them, *L. gasseri* has attracted increasing attention due to its reported ability to modulate the intestinal microbiota and exert antimicrobial, antioxidant, and immunomodulatory activities [27,28,29].

Studies integrating extraction parameters, the comparison of different drying methods and encapsulation systems, and the technological application of phenolic HS microcapsules in buttermilk-based fermented beverages remain limited. In this context, it was hypothesized that the composition of the encapsulating agents and the drying method would affect the retention and physicochemical characteristics of HS phenolic compounds, while the resulting microcapsules would preserve their functional properties after incorporation into a buttermilk-based fermented dairy beverage containing *L. gasseri*.

Therefore, the present study aimed to improve the extraction of phenolic compounds from HS using a food-grade solvent, microencapsulate the extracts by spray drying and freeze-drying using different encapsulating agents, characterize the resulting microcapsules, and evaluate their application in a buttermilk-based fermented dairy beverage containing *L. gasseri*. Overall, this study presents an integrated approach for the valorization of phenolic compounds from HS through extraction and microencapsulation processes, as well as its subsequent application in a sustainable fermented dairy matrix.

## 2. Materials and Methods

### 2.1. Chemical Products

All analytical-grade reagents were used without further purification. Trolox (6-Hydroxy-2,5,7,8-tetramethylchroman-2-carboxylic acid), 2,2-diphenyl-1-picrylhydrazyl (DPPH), 2,2′-azino-bis-(3-ethylbenzothiazoline-6-sulfonic acid) diammonium salt (ABTS), and 2,4,6-tris(2-pyridyl)-s-triazine (TPTZ) were purchased from Sigma-Aldrich^®^ (Burlington, MA, USA). Ethanol and food-grade cereal alcohol were obtained from ACS Científica^®^ (Sumaré, SP, Brazil). Maltodextrin (10 DE) was donated by Cargill^®^ (São Paulo, SP, Brazil), whereas inulin and whey protein concentrate (WPC) were supplied by Foodology, Univar Solutions^®^ (São Paulo, SP, Brazil). Folin–Ciocalteu reagent and gallic acid were obtained from Êxodo Científica^®^ (Sumaré, SP, Brazil). Sodium carbonate, aluminum chloride, sodium hydroxide, potassium chloride, sodium acetate, and all other reagents were of analytical grade.

### 2.2. Experimental Design

The experimental design consisted of three sequential stages.

In the first stage, the extraction conditions of phenolic compounds from HS calyces were evaluated by evaluating two food-grade solvents (ethanol and cereal alcohol) at three concentrations (50, 70, and 80%, *v*/*v*). The extract presenting the optimal amount of total phenolic compounds and antioxidant capacity was selected for the subsequent experiments.

In the second stage, the selected extract was microencapsulated using different wall material formulations composed of maltodextrin, inulin, and whey protein concentrate. The formulations were dried by freeze-drying and spray drying and subsequently characterized regarding their physicochemical properties, bioactive compound content, antioxidant capacity, and morphology. The formulation showing the best technological performance was selected for food application.

In the third stage, the selected microcapsules were incorporated into a buttermilk-based fermented dairy beverage containing *Lactobacillus gasseri*. The beverage was stored under refrigerated conditions for 28 days, and its physicochemical, microbiological, and functional stability was evaluated throughout storage.

### 2.3. Plant Material and Proximate Composition

Fresh calyces of HS were obtained from a local producer in Coimbra, Minas Gerais, Brazil, located in the Zona da Mata region of Minas Gerais, at an altitude of approximately 720 m (latitude: −20.830127 e longitude: −42.801351). The region has an average air temperature of 20.1 °C, an average relative humidity of 80.3%, and accumulated precipitation of approximately 1289.0 mm according to the National Institute of Meteorology (INMET). A voucher specimen was identified and deposited at the Herbarium of the Federal University of Viçosa under accession number VIC58246. The calyces were dried in a forced-air circulation oven at 40 °C until constant weight, ground using a knife mill, packed in airtight containers, and stored at −20 °C until use.

The proximate composition was determined according to AOAC [30]. Moisture was determined by oven drying, ash by incineration in a muffle furnace, protein content by the Kjeldahl method using a conversion factor of 6.25, lipid content by Soxhlet extraction with petroleum ether, and carbohydrate content was calculated by difference.

### 2.4. Extraction Parameters of Phenolic Compound

Extraction conditions were established based on preliminary trials involving the extraction of phenolic compounds from HS. Two types of solvent were evaluated: ethanol, as a reference chemical solvent, and food-grade cereal alcohol, selected as a suitable alternative for food applications. This comparison aimed to evaluate the extraction efficiency of the solvents and, simultaneously, to verify the feasibility of using a food-grade solvent to obtain an extract intended for subsequent application in a fermented dairy beverage for human consumption. Each solvent was evaluated at concentrations of 50, 70, and 80% (*v*/*v*), totaling six extraction conditions, as presented in Table 1.

Ground HS calyces were mixed with each solvent at a solid-to-solvent ratio of 1:10 (*w*/*v*). The mixtures were vortexed for 15 s to ensure complete dispersion of the plant material and subsequently shaken in an orbital shaker for 120 min at room temperature (approximately 25 °C).

After extraction, the suspensions were filtered through qualitative filter paper, and the solvents were removed, according to their initial concentration, under reduced pressure using a rotary evaporator with a water bath maintained at 40 °C. In this way, the ethanol or grain alcohol used during extraction was removed before subsequent analysis and processing steps. The concentrated extracts were transferred to amber flasks and stored at −80 °C until analysis. Total phenolic compounds, flavonoids, anthocyanins, and antioxidant capacity were determined for all extraction treatments. The extract presenting the best overall performance was selected for the microencapsulation experiments.

### 2.5. Microencapsulation of Phenolic Compounds

The extract selected in the previous stage was microencapsulated using different formulations composed of maltodextrin (MD), inulin (IN), and whey protein concentrate (WPC), according to Table 2.

The encapsulating formulations were prepared with a total solids concentration of 10% (*w*/*v*), according to the different proportions of inulin (IN), whey protein concentrate (WPC), and maltodextrin (MD) presented in Table 2. The concentration of inulin in the evaluated formulations ranged from 30% to 100%, being maintained at a minimum concentration of 30% in the combined formulations, with the aim of preserving the potential prebiotic functionality of the final ingredient. Thus, the composition of the encapsulating agents was one of the experimental factors evaluated, while the concentration of total solids (10%, *w*/*v*) was kept constant in all formulations.

The concentrated HS extract was incorporated into the encapsulating agent solution in a 1:5 (*v*/*v*) ratio, corresponding to one part extract (20 mL) and five parts encapsulating agent solution (100 mL), resulting in a final volume of 120 mL. This ratio and the volumes used were the same in all formulations. The mixtures were subjected to magnetic stirring until complete homogenization. The second experimental factor evaluated was the drying method, with each formulation subjected to lyophilization (FD) or spray drying (SD), as shown in Table 2. The other preparation conditions were maintained between treatments. The powders obtained were stored in laminated aluminum packaging, protected from light and moisture, at −20 °C until physicochemical characterization.

### 2.6. Drying of the Microcapsules

#### 2.6.1. Freeze-Drying

The feed dispersions prepared according to the formulations presented in Table 2 were distributed in stainless-steel trays and frozen at −80 °C for 24 h. Subsequently, the samples were freeze-dried in a bench-top freeze dryer (Terroni Equipamentos Científicos, Model LS3000, São Carlos, SP, Brazil) operating at approximately 0.05 mbar and a condenser temperature of −38 °C for 28 h. After drying, the powders were packed in laminated aluminum bags protected from light and moisture, and stored at −20 °C until physicochemical characterization.

#### 2.6.2. Spray Drying

The samples were subjected to spray drying in a mini spray dryer (Büchi B-290, Büchi Labortechnik AG, Flawil, Switzerland) equipped with a two-fluid nozzle (0.7 mm). The drying conditions were established based on preliminary trials to maximize powder recovery while minimizing moisture content, following the methodology described by Kurek et al. [9], with modifications. The operating parameters were as follows: inlet air temperature of 130 °C, outlet temperature ranging from 50 to 70 °C, feed flow rate of 2.0 mL min^−1^, drying air flow rate of 0.60 m^3^ min^−1^, and peristaltic pump rate of 6%. Compressed air was used as the atomizing gas according to the equipment operating conditions. After drying, the powders were immediately collected, packed in laminated aluminum bags, protected from light, and stored at −20 °C until further analyses.

#### 2.6.3. Encapsulation Efficiency

The encapsulation efficiency (*EE*) of total phenolic compounds was determined according to the equation below [31]:

$$EE\%=\frac{EPC}{MPC}\;\times \;100$$

where *EPC* is the initial mass of phenolic compounds in the extract (mg), calculated as:

*EPC* = *C_e_* × *V_e_*

*C_e_* is the concentration of phenolic compounds in the extract (mg/mL) and *V_e_* is the volume of extract used (mL) in microencapsulation. Afterwards, the *MPC* was calculated as follows, which is the mass of phenolic compounds recovered in the microcapsules (mg):

*MPC* = *C_m_* × *M_f_*

*C_m_* is the concentration of phenolic compounds in the microcapsules (mg/g) and *M_f_* is the total mass of recovered microcapsules (g). For each formulation, 20 mL of extract was combined with 100 mL of wall material solution (1:5, *v*/*v*). Thus, the measurements for the extract and the microcapsules were converted to the same basis (total mass of phenolic compounds) before calculating the *EE*. The dilution resulting from the addition of wall materials was accounted for by using the phenolic content expressed per gram of final microcapsules and the total mass of recovered microcapsules.

### 2.7. Physicochemical Characterization of the Microcapsules

The physicochemical characterization was carried out to compare the effects of the encapsulating agents and drying methods on the technological properties of the microcapsules. All analyses were performed in triplicate.

#### 2.7.1. pH and Total Soluble Solids

The pH of the microcapsule dispersions was determined using a digital pH meter (PG1000, Gehaka, São Paulo, SP, Brazil), previously calibrated with standard buffer solutions at pH 4.00 and 7.00. Total soluble solids (°Brix) were measured in the same dispersion using a digital refractometer (Lorben, São Paulo, SP, Brazil).

#### 2.7.2. Water Solubility Index

Water solubility was determined according to the methodology adapted from Anderson et al. [32]. Briefly, approximately 1 g of microcapsules was dispersed in distilled water at a ratio of 1:12 (*w*/*v*), homogenized under magnetic stirring and incubated at 37 °C. The suspensions were centrifuged at 3000× *g* for 20 min (Bunker Comercial Ltda., Model NT 815, Piracicaba, SP, Brazil). The supernatant was transferred to pre-weighed aluminum dishes and dried at 105 °C until constant weight.

Water solubility was calculated as follows:

$$WSI\;\left(\%\right)=\frac{MDS}{ISM}\;\times \;100$$

where *WSI* is the water solubility index (%), *MDS* is the mass of dissolved solids, and *ISM* is the initial sample mass.

### 2.8. Determination of Bioactive Compounds

Before analysis, the microcapsules were reconstituted in 70% (*v*/*v*) food-grade cereal alcohol, which had previously been selected as the extraction solvent due to its superior efficiency in recovering phenolic compounds, at a ratio of 1:13 (*w*/*v*), under magnetic stirring until complete dissolution. All analyses were performed in triplicate.

#### 2.8.1. Total Phenolic Compounds

Total phenolic compounds were determined using the Folin–Ciocalteu colorimetric assay according to Singleton and Rossi [33], with minor modifications. Briefly, 12.5 µL of sample were reacted with 12.5 µL of 20% Folin–Ciocalteu reagent for 3 min. Then, 25 µL of 7.5% sodium carbonate solution was added and left to stand for 60 min. The absorbances were then recorded at 760 nm using a microplate reader (Thermo Fisher Scientific, Multiskan GO, Vantaa, Finland). Results were expressed as mg gallic acid equivalents (GAE) per mL of extract or per gram of microcapsules.

#### 2.8.2. Total Flavonoids

Total flavonoids were determined using the aluminum chloride colorimetric method according to Zhishen et al. [34]. Briefly, 25 µL of samples were reacted with 100 µL of distilled water and 7.5 µL of 5% sodium nitrite solution for 6 min. Then, 7.5 µL of 10% aluminum chloride solution was added and left to stand for another 6 min. Finally, 100 µL of 4% sodium hydroxide solution and 10 µL of distilled water were added, and after 15 min, the absorbance was measured at 510 nm. Results were expressed as mg catechin equivalents (CE) per mL of extract or per gram of sample.

#### 2.8.3. Total Anthocyanins

Total monomeric anthocyanins were quantified using the pH differential method described by Giusti and Wrolstad [35]. Aliquots of samples were placed to react in potassium chloride (pH 1.0) and sodium acetate (pH 4.5) buffer for 15 min. Absorbance was measured at 510 and 700 nm for both buffer. Results were expressed as mg cyanidin-3-glucoside equivalents per mL of extract or per gram of sample.

### 2.9. Antioxidant Capacity

Antioxidant capacity was evaluated using ABTS and DPPH. All analyses were performed in triplicate.

#### 2.9.1. ABTS Assay

The ABTS•+ radical solution was prepared according to Re et al. [36]. For the microencapsulated samples, the samples were dissolved in the same solvent used to prepare the extract and subsequently centrifuged to remove insoluble material. The resulting supernatants were used for the assay. Blank samples were prepared and analyzed under the same experimental conditions to compensate for possible background absorbance from the encapsulating agentes. 50 µL of samples were reacted with 250 µL of ABTS radical, for 6 min. After reaction with the extracts, the absorbance was measured at 734 nm. A calibration curve was constructed using Trolox as the standard, and the antioxidant capacity of the samples was calculated from the resulting regression equation. Results were expressed as mmol Trolox equivalents per mL of the extract or gram of sample.

#### 2.9.2. DPPH Assay

DPPH radical scavenging activity was determined according to Re et al. [36]. Sample preparation and the use of blanks for the microencapsulated samples were performed as described in Section 2.9.1. 40 µL of sample was incubated with 300 µL of DPPH radical for 30 min. After incubation, absorbance was measured at 517 nm. A calibration curve was constructed using Trolox as the standard, and the antioxidant capacity of the samples was calculated from the resulting regression equation. Results were expressed as mmol Trolox equivalents (TE) per mL of the extract or gram of sample.

### 2.10. Scanning Electron Microscopy (SEM)

The morphology of the microcapsules was evaluated by SEM. The powders were fixed onto aluminum stubs using double-sided conductive carbon tape and sputter-coated with gold using a Quorum Q150R S sputter coater. Micrographs were obtained using a (JEOL Ltd., JSM-6010LA, Akishima, Tokyo, Japan) scanning electron microscope operating at an accelerating voltage of 12 kV. Representative images were recorded at different magnifications to evaluate particle morphology, surface integrity and structural differences between drying methods.

### 2.11. Development of a Model Buttermilk-Based Fermented Dairy Beverage

Following the physicochemical characterization of all microcapsule formulations, the spray-dried formulation containing 70% maltodextrin and 30% inulin was selected for incorporation into a model buttermilk-based fermented dairy beverage.

A model buttermilk-based fermented dairy beverage was developed to evaluate the technological feasibility of incorporating microencapsulated phenolic compounds from HS into a fermented dairy matrix. The beverage formulation was developed using buttermilk as the main dairy ingredient. Since the formulation is currently under intellectual property protection, the detailed composition of all ingredients will not be fully disclosed. However, all processing conditions were standardized to ensure the reproducibility of the technological application and subsequent physicochemical, microbiological, and functional evaluations.

Briefly, the dairy mixture (buttermilk and skimmed milk, 60% and 40%, respectively) was homogenized and subjected to heat treatment at 93 °C for 10 min, followed by cooling to 43 ± 1 °C. A commercial yogurt starter culture (Chr. Hansen A/S, Hørsholm, Denmark) was then added according to the manufacturer’s recommendations. Fermentation was carried out at 43 ± 1 °C until the pH reached 4.5, monitored using a calibrated digital pH meter. After fermentation, the product was cooled to 4 ± 1 °C and maintained under refrigeration for 12 h to allow structural stabilization. Subsequently, 5 g of the selected HS microcapsules were incorporated, followed by the addition of the strain *L. gasseri* LG08 (LEMMA Supply, São Paulo, SP, Brazil.) in lyophilized form, to ensure a minimum concentration of 10^9^ CFU. Both were incorporated aseptically under gentle agitation to ensure homogeneous distribution throughout the fermented dairy matrix. The beverage was then packaged in sterile polypropylene containers and stored under refrigerated conditions (4 ± 1 °C) for 28 days. The resulting beverage was used as an experimental model to evaluate the stability of microencapsulated phenolic compounds during refrigerated storage through physicochemical, microbiological, and antioxidant analyses.

#### 2.11.1. Storage Stability of the Fermented Dairy Beverage

The physicochemical, microbiological and functional stability of the fermented dairy beverage was evaluated throughout refrigerated storage. Analyses were performed on days 0, 7, 16 and 28 (T0, T7, T16 and T28, respectively). These storage times were selected to monitor possible changes in product quality throughout its expected shelf life. At each storage time, pH, soluble solids, titratable acidity, color parameters, microbiological quality, total phenolic compounds and antioxidant capacity were determined. All analyses were performed by triplicate.

#### 2.11.2. pH, Soluble Solids and Titratable Acidity

The pH was measured directly using a calibrated digital pH meter (PG1000, Gehaka, Brazil). Total soluble solids (°Brix) were determined using a digital refractometer (Lorben^®^). Titratable acidity was determined according to AOAC [30] by titration with standardized 0.1 mol L^−1^ sodium hydroxide solution using phenolphthalein as the endpoint indicator. Results were expressed as g of lactic acid per 100 g of sample.

#### 2.11.3. Color Measurement

Color was evaluated using the CIELab color system using a Colour Quest II colorimeter (Hunter Lab, Reston, VA, USA), connected to a computer equipped with the Universal software system V4.10 (ColorQuest XE, Reston, EUA). The equipment was properly calibrated for included reflectance, using an observer angle of 10° and D65 illuminant, with the positioning of the light protection devices, calibrated white tile and calibrated grey tile on the reflectance port. The parameters L* (lightness), a* (red-green coordinate) and b* (yellow-blue coordinate) were recorded. Chroma (C*), Hue angle (*h*°) and total color difference (Δ*E*) were calculated according to the following equations:

$$h{}^{\circ}=\arctan\left(\frac{b^{*}}{a^{*}}\right)$$

$$C^{*}={\left[{\left(a^{*}\right)}^{2}+{\left(b^{*}\right)}^{2}\right]}^{1/2}$$

where *a* is the value of chromatic coordinate *a* and *b* is the value of chromatic coordinate *b*.

$${∆E}^{*}\;=\;{[{\left({∆L}^{*}\right)}^{2}+{\left({∆a}^{*}\right)}^{2}+{\left({∆b}^{*}\right)}^{2}]}^{1/2}$$

where Δ*E* is the total color difference, Δ*L* is the change in the L* coordinate, Δ*a* is the change in the a* coordinate, and Δ*b* is the change in the b* coordinate.

Three readings were obtained for each sample and the average value was considered for statistical analysis.

#### 2.11.4. Microbiological Analyses

The microbiological quality of the fermented beverage was evaluated by determining lactic acid bacteria (LAB) counts and total bacteria count (TBC). LAB counts were performed using de Man, Rogosa and Sharpe (MRS) agar after serial decimal dilution of the samples. The plates were incubated anaerobically at 37 ± 2 °C for 48 h. Results were expressed as log CFU g^−1^. Total aerobic mesophilic bacteria were determined using Plate Count Agar (PCA) according to APHA recommendations [37]. All microbiological analyses were carried out in duplicate.

#### 2.11.5. Kinetics of Total Phenolic Compound Stability

The stability of total phenolic compounds was monitored in the fermented dairy beverage throughout 28 days of storage. Analyses were performed in triplicate at four sampling points: immediately after preparation (T0) and after 7 (T7), 16 (T16), and 28 (T28) days of storage.

The degradation kinetics of total phenolic compounds were determined based on changes in the total phenolic compound content over the storage period [31]. The degradation rate constant (k) and half-life (t_1_/_2_), defined as the time required for a 50% reduction in the initial phenolic compound content, were calculated using the equations below:

$$In\;\left(\frac{C_{t}}{C_{0}}\right)=-k\;\times \;t$$

$$t\frac{1}{2}=-In\;0.5\times k^{-1}$$

where (C_0_) represents the initial total phenolic compound content, (C_t_) is the content determined after (t) days of storage, and (k) is the degradation rate constant.

### 2.12. Phenolic Compound Profiling and Quantification by Ultra-High-Performance Liquid Chromatography–Tandem Mass Spectrometry (UPLC-MS/MS)

The phenolic compound profile was analyzed in dehydrated HS calyces, HS extract microcapsules, and the fermented dairy beverage. This extraction procedure was specifically adapted to the requirements of UPLC-MS/MS analysis to ensure adequate recovery and minimize potential matrix interference. For extraction, approximately 110 mg of each sample were macerated in liquid nitrogen and subjected to extraction with 400 μL of methanol:isopropanol:acetic acid (20:79:1, *v*/*v*/*v*). The samples were vortexed, sonicated for 10 min, kept on ice for 30 min, and sonicated again for 10 min. After centrifugation (13,000× *g*, 10 min, 4 °C), the supernatants were collected and the extraction procedure was repeated once with the residual pellet. The supernatants were combined, centrifuged at 20,000× *g* for 5 min at 4 °C, and filtered through a 0.2 μm PVDF membrane.

The identification and quantification of phenolic compounds were performed at the Núcleo de Análises de Biomoléculas (NuBioMol/UFV), using an Agilent 1200 Infinity Series system coupled to a triple quadrupole mass spectrometer (QqQ 6430: Agilent Technologies, Inc., 6430 Triple Quadrupole LC/MS, Santa Clara, CA, USA). The samples (20 μL) were analyzed using a Hypersil™ ODS C18 column (3 μm, 2.1 × 100 mm; Thermo Fisher Scientific Inc., Waltham, MA, USA), coupled to a Zorbax SB-CN guard column (1.8 μm, 3.0 × 5 mm; Agilent Technologies, Inc., Santa Clara, CA, USA). Chromatographic separation was performed using water containing 0.02% acetic acid (A) and acetonitrile containing 0.02% acetic acid (B), under a gradient program of 2% B at 0 min, 20% B at 8 min, 40% B at 15 min, 98% B at 18 min, 98% B at 21 min, 2% B at 22 min, 2% B at 23 min, and 2% B at 25 min, with a flow rate of 0.3 mL/min and a column temperature of 23 °C. The total injection run time was 25 min. Detection was performed by electrospray ionization (ESI) in positive and negative modes, using multiple reaction monitoring (MRM). Absolute quantification was performed using external calibration curves (0.5–1000 ng), using the respective analytical standards. Data were processed using Skyline software version 26.1, and the results were expressed as mg/g of HS calyces, mg/5 g of microcapsules, and mg/100 mL of fermented dairy beverage, corresponding to the raw material used to obtain the extract, the amount of microcapsules incorporated into the formulation, and the amount of beverage produced, respectively.

### 2.13. Statistical Analysis

The experimental data were analyzed using GraphPad Prism^®^ version 8.0 (GraphPad Software Inc., San Diego, CA, USA).

Data were expressed as mean ± standard deviation. Initially, results from the analyses of extracts and microcapsules were evaluated using two-way analysis of variance (ANOVA) followed by Tukey’s multiple comparison test, as the data met the assumptions of normality. For the storage stability study, since the data did not meet the assumptions for parametric analysis, differences between storage periods were evaluated using the Kruskal–Wallis test followed by Dunn’s multiple comparison test. The degradation constant (k) and half-life (t½) were determined via regression analysis.

## 3. Results and Discussion

### 3.1. Proximate Composition of HS

**Figure 1:**
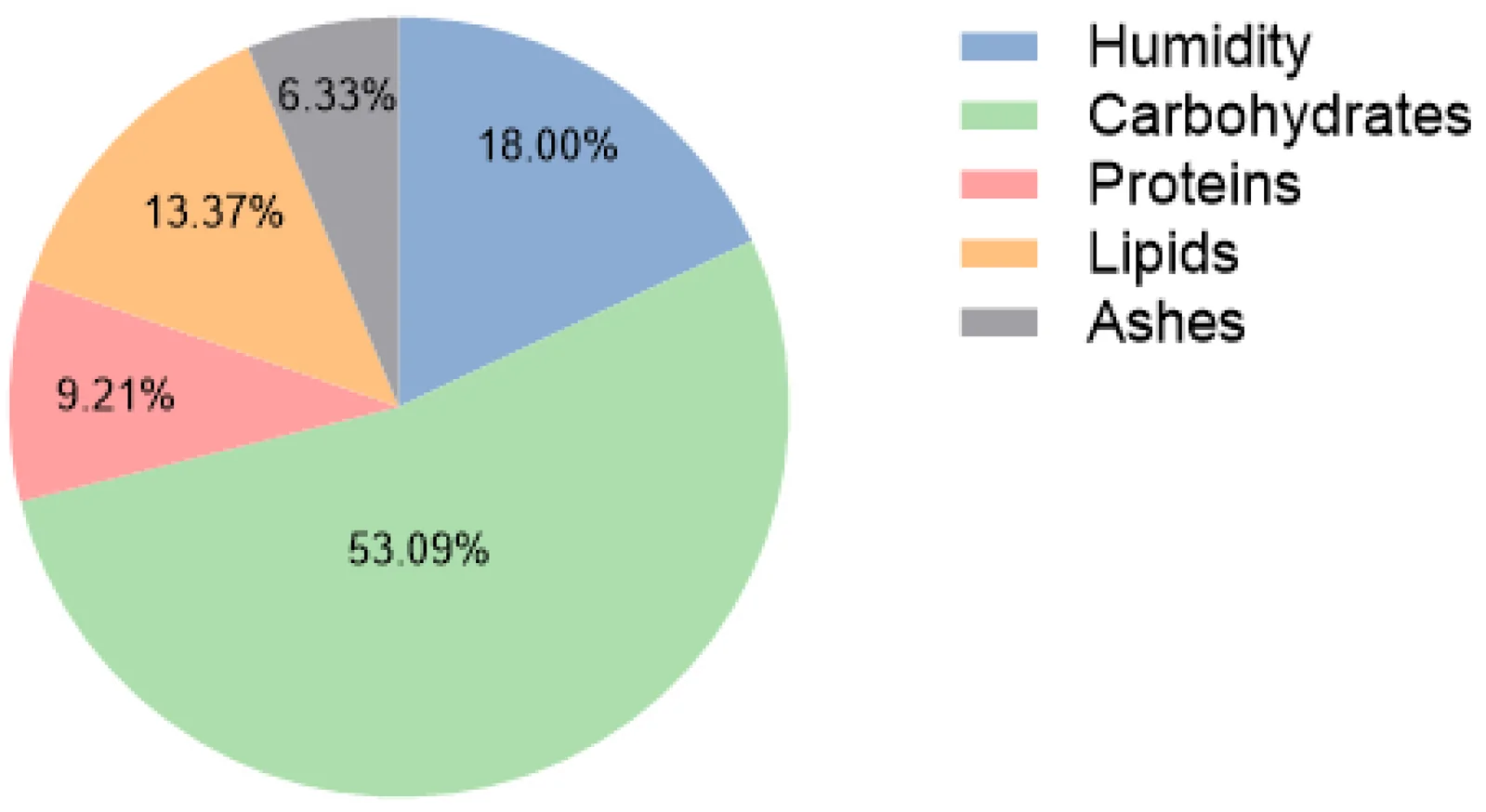
Proximate composition of dehydrated *Hibiscus sabdariffa* calyces.

The proximate composition of the dehydrated calyces of HS is presented in Figure 1.

Carbohydrates were the predominant component, followed by moisture, lipids, proteins, and ash, confirming the characteristic composition of this species reported in previous studies [38,39]. Variations in the chemical composition of HS have been associated with factors such as cultivar, climatic conditions, agricultural practices, harvest stage, and storage conditions [39]. The characterization of the raw material provides important information for standardizing the extraction process and supports the subsequent evaluation of phenolic extraction, microencapsulation, and application in the fermented dairy beverage.

### 3.2. Effect of Solvent Type and Concentration on the Extraction of Total Phenolic Compounds from HS

Phytochemicals are present in low concentrations in plant matrices, and their efficient extraction is essential to maximize the recovery of bioactive compounds and enable the evaluation of their functional properties [9,40]. The extraction process is influenced by several factors, including solvent type, concentration, extraction time, temperature, and pH [6,41]. Solvent composition can influence extraction efficiency, as the combination of water with organic solvents can facilitate solvent penetration into the plant matrix and increase the solubility of phenolic compounds with varying polarities [42].

In the present study, the extraction of phenolic compounds from HS was compared using ethanol and grain alcohol at different concentrations. Overall, the 70% grain alcohol extract (E5) showed higher concentrations of total phenolic compounds, total flavonoids, and total anthocyanins, as well as higher antioxidant capacity, compared to the other extracts (Table 3).

Among the ethanol extracts, the 70% concentration (E2) showed higher values for bioactive compounds and antioxidant capacity compared to extracts E1 and E3. Specifically, the concentration of total phenolic compounds was higher in E5 and E6, with no significant difference between them. Similarly, E5 and E6 demonstrated higher levels of total flavonoids, total anthocyanins, and antioxidant capacity (ABTS), with no differences between them. Only in the DPPH assay was E5 significantly higher than E6. Therefore, these extracts (E5 and E6) exhibited the highest observed antioxidant capacity. In contrast, E3, corresponding to the 80% ethanol extract, generally showed lower concentrations for all evaluated parameters. These results suggest that grain alcohol at concentrations of 70% and 80% favored the recovery of bioactive compounds. The flavonoid group includes anthocyanins, which are particularly relevant in HS, as they are the primary pigments responsible for its coloration. Although E5 and E6 showed no statistical difference, E5 was selected for the microencapsulation stage because it exhibited, in descriptive terms, the highest values for the evaluated compounds and antioxidant capacity, in addition to using a lower proportion of organic solvent compared to E6.

The superior performance of the 70% hydroalcoholic solution can be attributed to its intermediate polarity, which facilitates the swelling of plant tissues, favors mass transfer, and promotes the solubilization of phenolic compounds [42]. Therefore, the extraction conditions employed, including solvent composition and the extraction technique, can influence the recovery of phenolic compounds [6,40].

From a technological perspective, the superior performance of cereal alcohol is particularly relevant because it is a food-grade solvent, allowing the direct application of the extract in food systems without restrictions associated with non-food-grade solvents. Therefore, besides improving extraction efficiency, the use of cereal alcohol represents an important advantage for the development of functional foods containing natural bioactive compounds.

**Figure 2:**
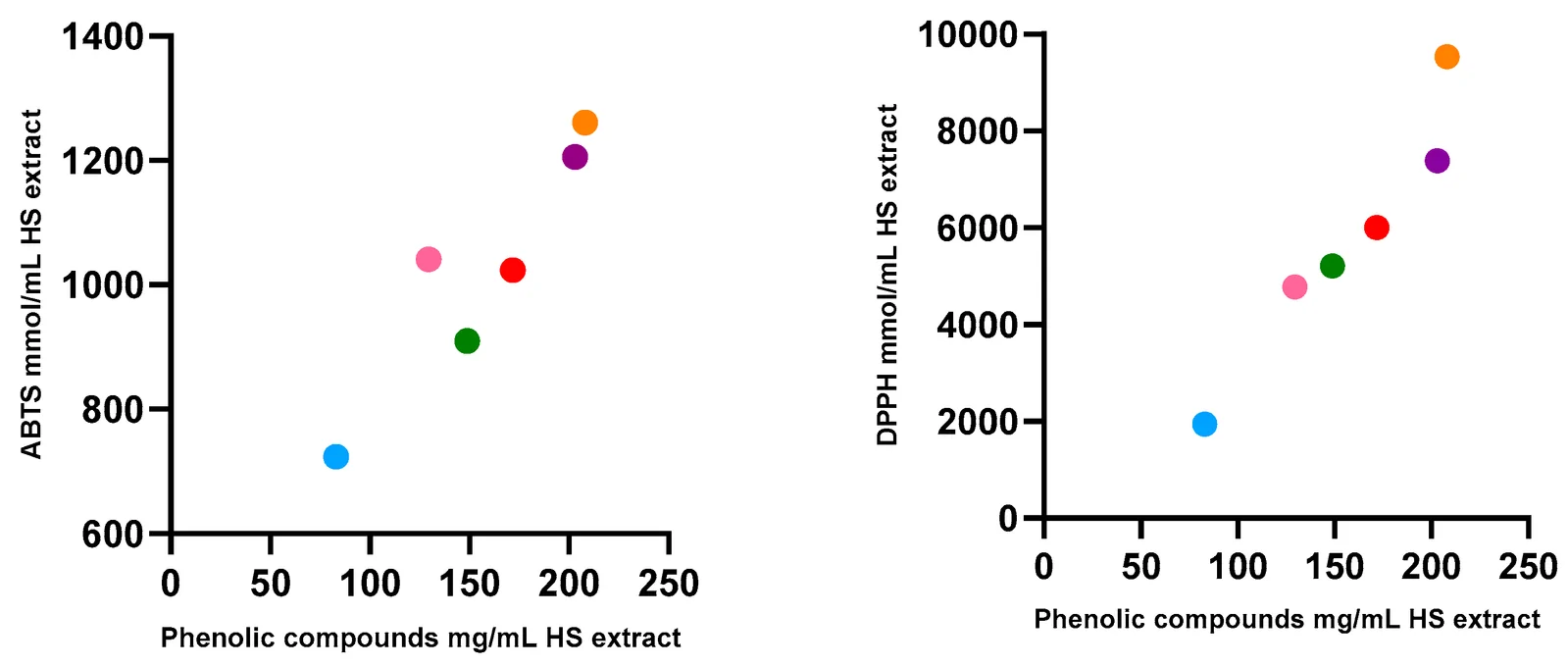
Pearson correlation between the total phenolic compound concentration of HS extracts and the antioxidant capacity determined by the ABTS and DPPH assays in HS extracts. The correlation coefficients were *r* = 0.85 for ABTS and *r* = 0.92 for DPPH.

Regarding the antioxidant properties of the extracts, phenolic compounds are widely recognized for their ability to scavenge free radicals [42]. Figure 2 shows a positive correlation between the total phenolic content of the extracts and their antioxidant capacity determined by the ABTS and DPPH assays. This result indicates that higher concentrations of phenolic compounds increase the availability of hydroxyl groups in the reaction medium, enhancing hydrogen donation and consequently promoting the neutralization of free radicals [40].

Phenolic compounds possess recognized antioxidant capacity, contributing to the neutralization of reactive species [43,44,45]. Thus, selecting appropriate extraction conditions is important not only to enhance the recovery of these compounds but also to obtain extracts with greater antioxidant potential for subsequent microencapsulation and application in functional food systems.

### 3.3. EE%, °Brix, pH, and Solubility of the Microcapsules

**Figure 3:**
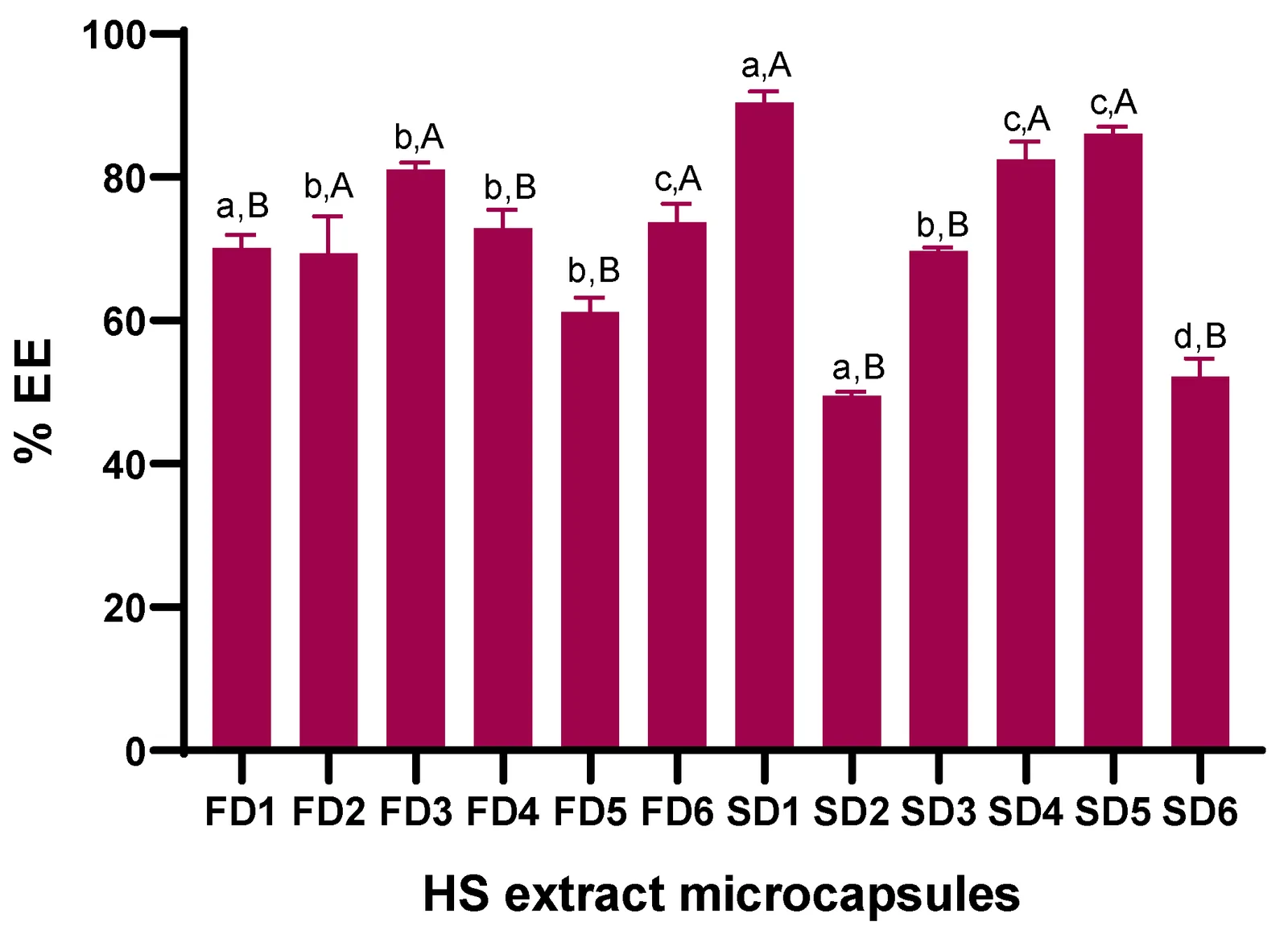
Encapsulation efficiency of phenolic compounds in microcapsules obtained by freeze-drying (FD1–FD6) and spray-drying (SD1–SD6). Values are expressed as mean ± standard deviation. Different lowercase letters indicate significant differences among formulations subjected to the same drying method, while different uppercase letters indicate significant differences between drying methods for the same formulation, according to ANOVA and Tukey’s test (*p* < 0.05).

The results indicated significant differences between the formulations and the drying methods (Figure 3).

Among the freeze-dried formulations, FD3 showed the highest value, while FD5 showed the lowest. For the spray-dried samples, SD2 and SD6 exhibited the highest efficiencies, whereas SD1 and SD4 showed the lowest values. When comparing the drying methods, higher efficiencies were observed with spray drying for formulations SD1, SD4, and SD5, while freeze-drying resulted in higher efficiencies for FD2, FD3, and FD6. These results suggest that encapsulation efficiency can be influenced by both the composition and proportion of the encapsulating agents and the drying method, given that different combinations of agents and methods exhibited distinct behaviors.

The physicochemical properties of the microcapsules, including °Brix, pH, and water solubility, are important indicators of their stability and technological applicability in food systems.

The mean °Brix value was 75% for the microcapsules containing HS phenolic compounds. The high concentration of soluble solids may contribute to reducing water availability, thereby improving the stability of the microcapsules and favoring the preservation of bioactive compounds during storage [11].

Regarding pH (Table 4), all microcapsule formulations exhibited an acidic character, which can be attributed to the HS extract, naturally acidic in nature [46]. This characteristic is considered advantageous because the stability of anthocyanins, the main pigments present in HS, is enhanced under acidic conditions, in which the flavylium cation structure remains more stable. Consequently, the acidic environment contributes to preserving color intensity and antioxidant properties, supporting the use of anthocyanins as both natural colorants and bioactive compounds with potential functional effects [47].

**Figure 4:**
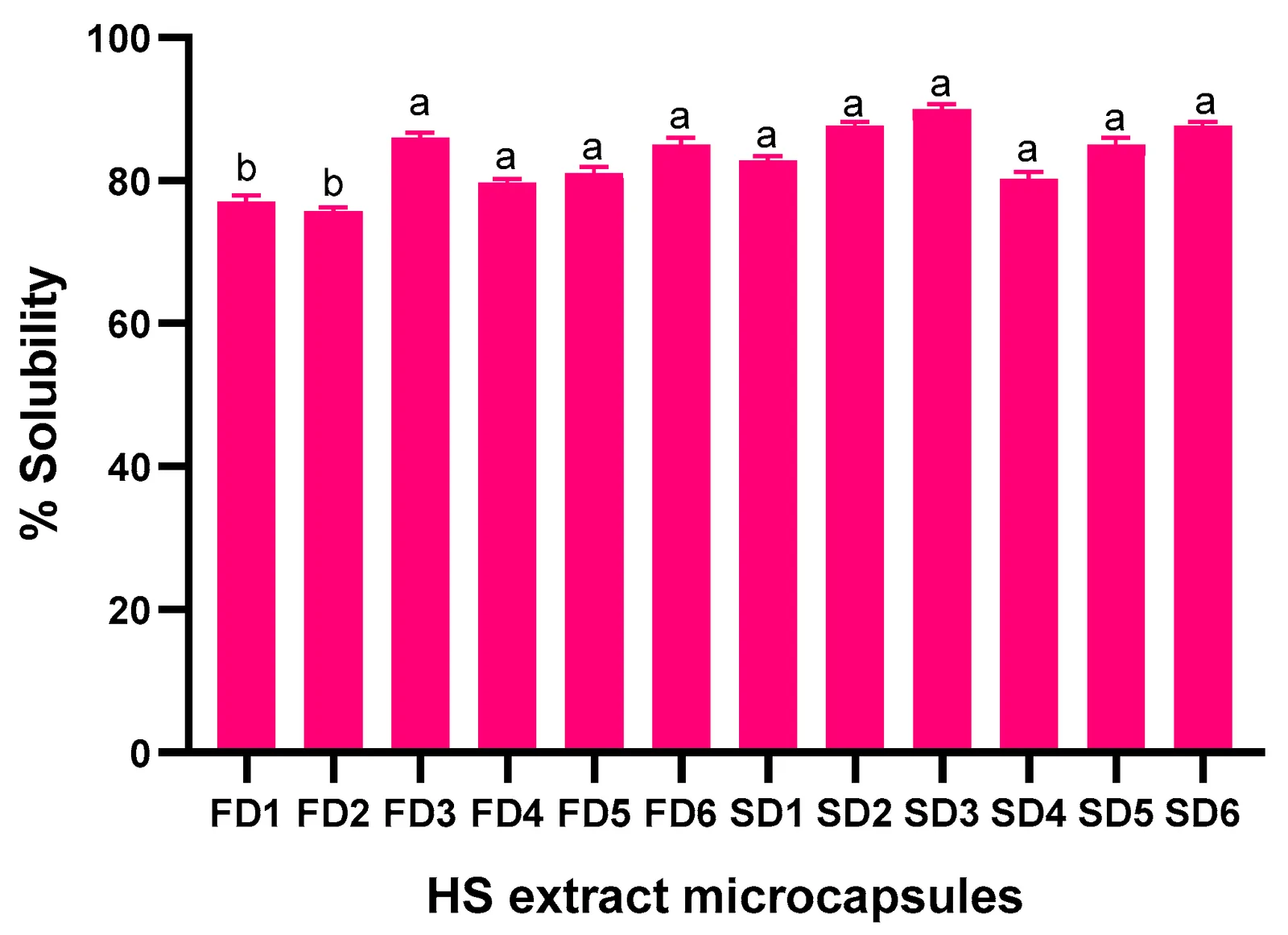
Solubility (%) of HS phenolic compound microcapsules obtained by freeze-drying (FD) and spray drying (SD) using different encapsulating agents: FD1 and SD1 (100% inulin); FD2 and SD2 (30% inulin + 70% concentrated whey protein); FD3 and SD3 (30% inulin + 70% maltodextrin); FD4 and SD4 (65% inulin + 35% concentrated whey protein); FD5 and SD5 (65% inulin + 35% maltodextrin); FD6 and SD6 (30% inulin + 35% maltodextrin + 35% concentrated whey protein). Different lowercase letters above the bars indicate statistically significant differences between treatments (Kruskal–Wallis test followed by Dunn’s multiple-comparison test, *p* < 0.05).

Another parameter evaluated was water solubility, an essential technological property for powdered ingredients intended for incorporation into foods and beverages, as it directly influences dispersibility, reconstitution, and product homogeneity [48]. All microcapsule formulations containing HS phenolic compounds (Figure 4) exhibited solubility values above 75%, indicating good reconstitution properties regardless of the drying method. Among the evaluated formulations, SD3 exhibited the highest solubility (>90%), which can be attributed to the combination of 30% inulin and 70% maltodextrin.

**Figure 5:**
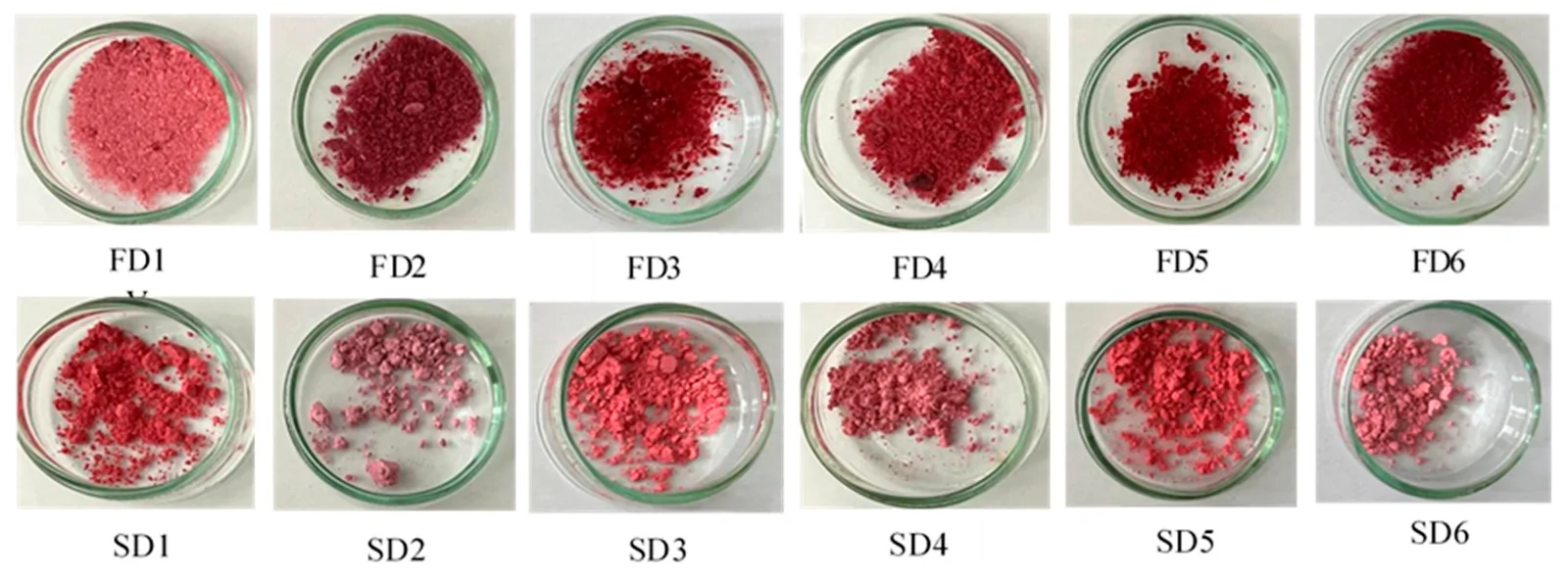
HS phenolic compound obtained using two drying methods, freeze-drying (FD) and spray drying (SD), with different encapsulating agents: FD1 and SD1 (100% inulin); FD2 and SD2 (30% inulin + 70% concentrated whey protein); FD3 and SD3 (30% inulin + 70% maltodextrin); FD4 and SD4 (65% inulin + 35% concentrated whey protein); FD5 and SD5 (65% inulin + 35% maltodextrin); FD6 and SD6 (30% inulin + 35% maltodextrin + 35% concentrated whey protein).

The high solubility observed for SD3 can be explained by the physicochemical characteristics of the selected encapsulating agents. Maltodextrin is a highly hydrophilic carrier with excellent water solubility, while its interaction with inulin through intermolecular hydrogen bonding enhances particle hydration and confirms the synergistic effect of combining encapsulating agentes [48,49]. Furthermore, the spray-drying process contributed to increased solubility, as the resulting powders exhibited finer and more homogeneous particles (Figure 5), increasing the contact surface with water and facilitating their dispersion and incorporation into different food matrices.

From a technological perspective, these findings demonstrate that both the encapsulating system and the drying method directly influenced the functional properties of the microcapsules. The superior solubility and more homogeneous morphology obtained for the formulation SD3 containing 70% maltodextrin and 30% inulin produced by spray drying justified its selection for application in the fermented dairy beverage, reinforcing its potential for industrial application.

### 3.4. Quantification of Bioactive Compounds and Antioxidant Capacity of the Microcapsules

The microcapsules were produced using different combinations of encapsulating agents and subjected to two drying methods, freeze-drying and spray drying. For comparative purposes, identical formulations were processed by both techniques and subsequently characterized regarding their bioactive compound content and antioxidant capacity.

The contents of total phenolic compounds, total flavonoids, and total anthocyanins, as well as the antioxidant capacity of the microcapsules, are presented in Table 5. Regarding total phenolic compounds, significant differences were observed among the formulations within both drying methods. Among the freeze-dried formulations, FD3 exhibited the highest concentration of these compounds, whereas among the spray-dried formulations, SD1 and SD5 showed the highest values. Furthermore, differences between the drying methods were observed for formulations 3 and 6, with higher contents in FD3 and FD6, respectively.

For total flavonoids, no significant differences were observed among the freeze-dried formulations; however, among the spray-dried formulations, SD2, SD4, and SD6 showed the highest contents. Comparison between the methods revealed a significant difference only for formulation 5, with a higher content in FD5. Regarding total anthocyanins, significant differences were observed among the formulations in both drying methods. Among the freeze-dried samples, FD2, FD3, FD5, and FD6 exhibited the highest contents, while SD2, SD5, and SD6 showed the highest values among the spray-dried samples. When comparing the methods, significant differences were observed for formulations 3 and 4, with higher concentrations in FD3 and SD4, respectively.

Antioxidant capacity also varied among the formulations. For the ABTS assay, FD6 showed the highest value among the freeze-dried formulations, while SD2 showed the lowest value among the spray-dried formulations. Differences between the methods were observed for formulations 2, 4, 5, and 6, with higher values in the freeze-dried samples. For the DPPH assay, FD4 exhibited the highest antioxidant activity among the freeze-dried formulations, while FD3 exhibited the lowest. Among the spray-dried samples, SD1 and SD2 showed the highest values. Differences between the methods were observed in formulations 2, 4, and 6, with higher values also found in the freeze-dried samples.

Overall, the results indicate that the drying method and the composition of the encapsulating matrix influenced the retention of bioactive compounds and the antioxidant activity of the microcapsules. Freeze-drying performed better for some of the evaluated parameters; this may be attributed to the milder process conditions, specifically the low temperatures employed, which can favor the preservation of heat-sensitive bioactive compounds [50,51]. However, spray drying stands out due to its technological and economic advantages, including shorter processing time, continuous operation, and greater suitability for large-scale industrial production [50,52]. Furthermore, this technique is widely used for the encapsulation of heat-sensitive compounds because the rapid evaporation of water during the process minimizes thermal degradation while maintaining product quality [53,54]. These findings support the selection of spray drying for the production of HS microcapsules intended for application in functional foods.

### 3.5. Morphology of Microcapsules by Scanning Electron Microscopy (SEM)

**Figure 6:**
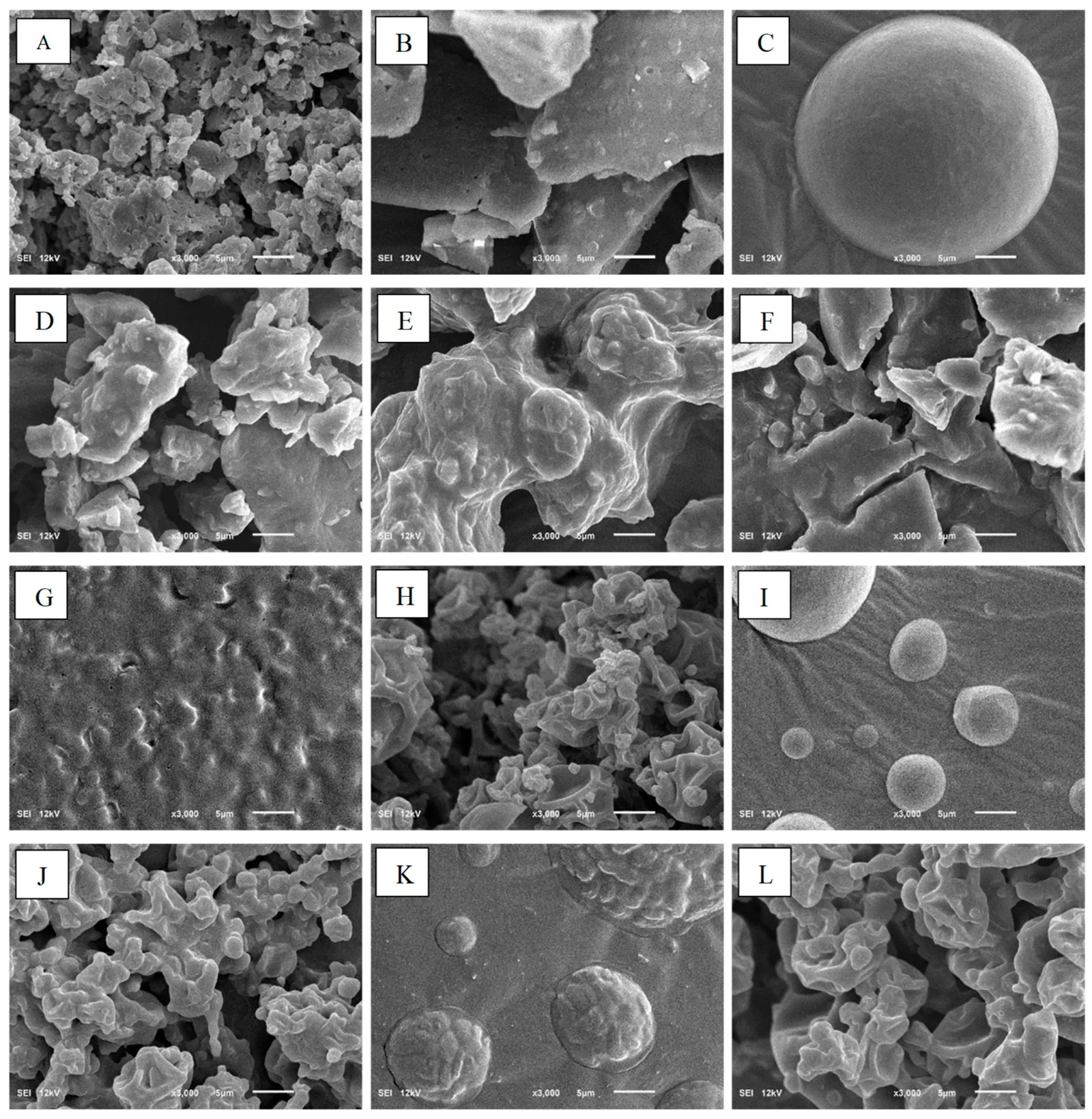
Scanning electron microscopy images (3000× magnification) of *Hibiscus sabdariffa* extract microcapsules dried by freeze-drying (**A**–**F**) and spray drying (**G**–**L**) with different encapsulating agents: (**A**,**G**) (100% inulin); (**B**,**H**) (30% inulin + 70% concentrated whey protein); (**C**,**I**) (30% inulin + 70% maltodextrin); (**D**,**J**) (65% inulin + 35% concentrated whey protein); (**E**,**K**) (65% inulin + 35% maltodextrin); (**F**,**L**) (30% inulin + 35% maltodextrin + 35% concentrated whey protein).

The morphology of the microcapsules containing HS extract obtained by freeze-drying and spray drying is presented in Figure 6.

The micrographs demonstrated that both the drying method and the encapsulating system markedly influenced particle morphology. Freeze-dried microcapsules exhibited irregular, porous, and collapsed structures, whereas spray-dried microcapsules showed predominantly spherical particles with fewer structural deformations. These morphological differences are expected because freeze-drying removes water by sublimation, generating porous matrices, while spray drying promotes rapid water evaporation, favoring the formation of compact and spherical particles [49].

Variations in particle size and surface texture were also observed among the formulations, corroborating previous findings reported by Ferreira et al. [55]. Particle sizes ranged approximately from 3 to 40 µm, as estimated from the micrographs. However, due to the high polydispersity and irregular particle morphology, particularly in the freeze-dried samples, a representative mean particle size could not be reliably determined. Such differences are directly associated with the physicochemical properties of the wall materials, including viscosity, film-forming ability, and drying behavior, which determine the formation and integrity of the particle wall during solvent removal [49].

The encapsulating system played a decisive role in determining particle morphology. Formulations containing maltodextrin produced microcapsules with smoother surfaces and greater structural homogeneity [17]. In particular, the formulation containing 70% maltodextrin and 30% inulin produced by spray drying (Figure 6I) generated spherical particles without visible cracks or structural collapse. Maltodextrin exhibits low viscosity, high water solubility, and excellent film-forming capacity, characteristics that favor the rapid formation of a continuous matrix during spray drying and reduce structural defects [17,55,56]. These morphological characteristics are consistent with the higher water solubility (>90%) observed for this formulation, supporting its selection for incorporation into the fermented dairy beverage.

Conversely, microcapsules prepared with inulin as the sole wall material (Figure 6A,G) exhibited rougher surfaces and more irregular structures. This behavior is probably associated with the hygroscopic and fibrous nature of inulin, whose polymeric chains establish extensive hydrogen bonding with water molecules, impairing the formation of a continuous wall during drying [49]. Although inulin contributes prebiotic functionality, its use as the sole encapsulating agent may compromise the technological quality of the resulting particles.

The addition of whey protein concentrate (WPC) also affected particle morphology. Due to its amphiphilic characteristics and high protein content, WPC improves emulsion stability and forms cohesive interfacial films through hydrophobic interactions and hydrogen bonding with phenolic compounds [57,58]. Accordingly, formulations containing 35% (Figure 6D,J) or 70% WPC (Figure 6B,H) produced by spray drying exhibited predominantly spherical particles with continuous surfaces, although the simultaneous presence of inulin slightly increased surface roughness.

Overall, the SEM results reinforce the physicochemical findings obtained in this study, demonstrating that the combination of spray drying with 70% maltodextrin and 30% inulin generated microcapsules with superior technological characteristics, including homogeneous morphology, high water solubility, and absence of structural defects. These characteristics are particularly desirable for food applications because they facilitate powder dispersion, improve physical stability, protect encapsulated phenolic compounds, and favor the incorporation of the microcapsules into dairy matrices.

### 3.6. Physicochemical Characteristics, Microbiological Quality, Phenolic Compounds, Antioxidant Capacity, Color Stability, and Phenolic Degradation Kinetics of the Beverage During Refrigerated Storage

To demonstrate the technological applicability of the microencapsulated phenolic compounds from HS, a buttermilk-based fermented dairy beverage was developed using the selected microcapsules as a natural colorant and functional ingredient. The stability of the physicochemical, microbiological, functional, and color characteristics was monitored during 28 days of refrigerated storage.

**Figure 7:**
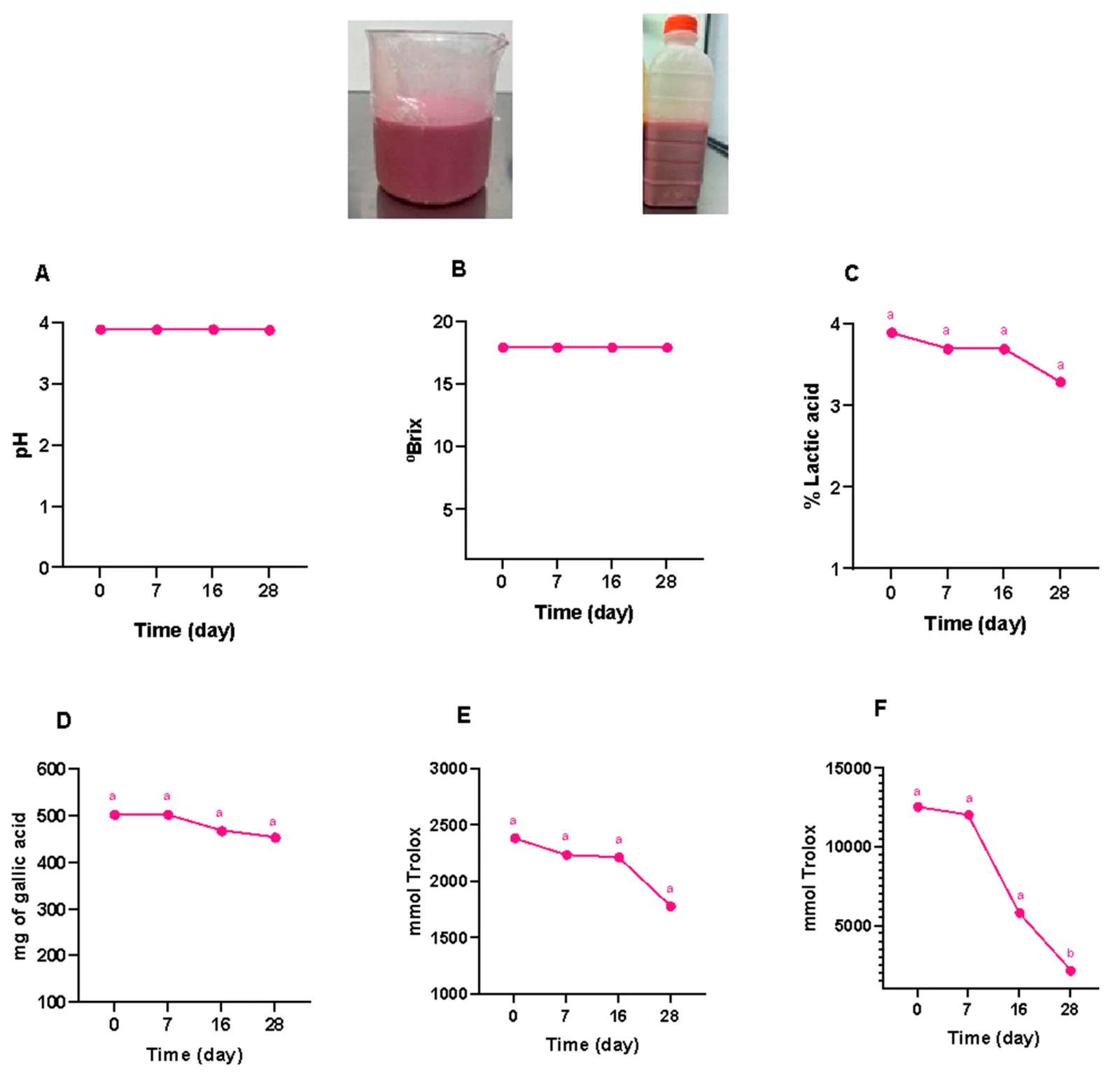
Variation in pH (**A**), total soluble solids (**B**), titratable acidity (**C**), total phenolic compounds (**D**), and antioxidant capacity determined by the ABTS (**E**) and FRAP (**F**) assays of a fermented dairy beverage based on buttermilk incorporated with microencapsulated phenolic extract from *Hibiscus sabdariffa* L. during 28 days of storage at 8 °C. Different lowercase letters indicate significant differences (*p* < 0.05) between each storage time and the initial time (day 0), according to ANOVA followed by Dunnett’s multiple comparisons test.

The physicochemical characteristics of the beverage remained stable throughout storage (Figure 7).

No differences were observed in pH, total soluble solids, or titratable acidity among the evaluated storage times (0, 7, 16, and 28 days), indicating that the beverage matrix remained stable under refrigerated conditions. Similarly, the concentration of total phenolic compounds did not change during storage, demonstrating that the microencapsulation process contributed to the stability of the phenolic compounds during storage. This result reinforces the protective effect previously observed for the microcapsules and confirms their suitability for incorporation into fermented dairy products.

Regarding antioxidant capacity, the ABTS assay did not show differences among storage periods, whereas the antioxidant activity determined by the FRAP assay decreased at day 28. These differences can be explained by the distinct reaction mechanisms of the two assays. While ABTS primarily measures radical scavenging capacity, FRAP evaluates the reducing power of antioxidant compounds. Therefore, subtle structural modifications of phenolic compounds during storage may have reduced their electron-donating capacity without substantially affecting their free radical scavenging activity. This interpretation is consistent with the stable total phenolic content observed throughout storage, indicating that changes were associated with the antioxidant behavior of specific compounds rather than with an overall loss of phenolics.

**Figure 8:**
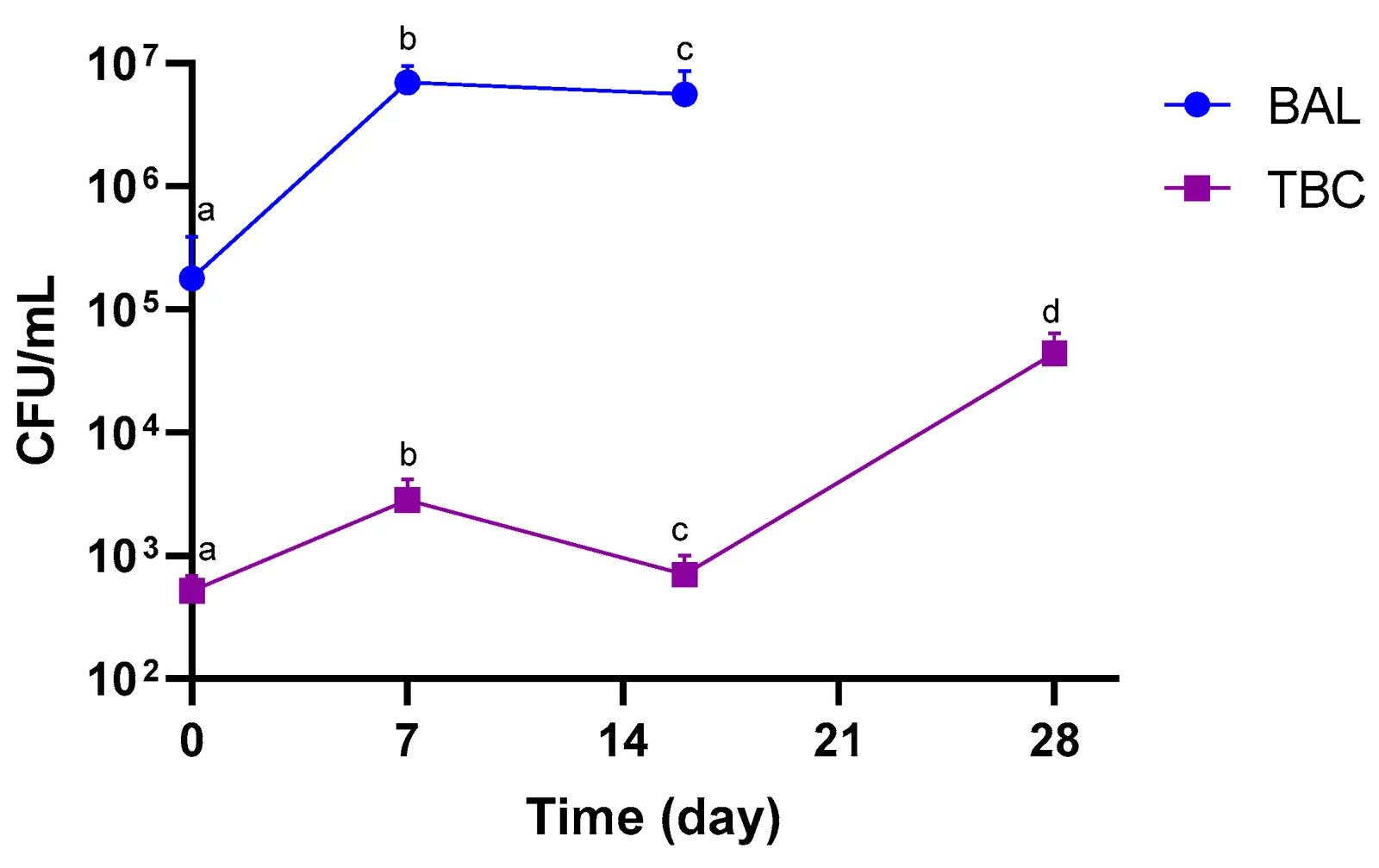
Total bacterial count (TBC) and lactic acid bacteria count (LAB) of a buttermilk-based fermented dairy beverage incorporated with microencapsulated phenolic extract from *Hibiscus sabdariffa* L. during 28 days of storage at 8 °C. Different lowercase letters indicate significant differences (*p* < 0.05) between each storage time and the initial time (day 0), according to ANOVA followed by Dunnett’s multiple comparisons test.

The viability of LAB (Figure 8) increased during the first seven days of refrigerated storage, indicating successful microbial adaptation and metabolic activity in the fermented beverage. After 16 days, LAB counts gradually declined, whereas TBC increased at the end of storage. Because no significant changes were observed in pH or titratable acidity during storage, the reduction in LAB viability cannot be attributed solely to acidification of the product. Instead, it is more likely associated with nutrient depletion during prolonged storage, accumulation of microbial metabolites, and competition with other microorganisms, as suggested by the increase in TBC [59,60]. Although TBC increased at the end of storage, this result should not necessarily be interpreted as microbial spoilage, since the assay quantifies the total cultivable aerobic microbiota and does not differentiate between beneficial and deteriorative microorganisms. Additional microbiological identification would be necessary to determine the composition of this microbial population.

The color parameters (L*, a*, b*, C*, h°, and ΔE) are presented in Table 6. Initially, the beverage exhibited a predominantly reddish color with a minor contribution from yellow tones, a characteristic consistent with the presence of natural HS pigments. During storage, changes in color parameters relative to the initial condition were observed, including a reduction in lightness (L*) and redness (a*), accompanied by an increase in yellowness (b*) and hue angle (h°). This behavior indicates a gradual shift in color from reddish tones toward more yellowish hues. Additionally, the reduction in chroma (C*) suggests a decrease in color saturation, while the progressive increase in ΔE highlights cumulative color changes throughout the storage period. ΔE values rose from 3.07 on day 7 to 10.00 on day 28, indicating perceptible color differences based on established perception thresholds [61]. Taken together, these results demonstrate a progressive change and a reduction in the beverage’s color intensity during storage.

Importantly, these color changes cannot be attributed to a general degradation of phenolic compounds, since total phenolic content remained stable throughout storage. Instead, they are more likely related to structural transformations of anthocyanins, including hydration, copigmentation, and interactions with milk proteins, which alter color expression without necessarily changing the overall concentration of phenolic compounds [35]. Similar interactions between anthocyanins and dairy proteins have been reported to affect color stability while preserving the phenolic content of dairy products. This interpretation is also supported by the maintenance of ABTS antioxidant activity throughout storage, indicating that the functional properties of the beverage were largely preserved despite the observed color modifications. Furthermore, the reduction in the L* parameter may also be associated with proteolytic activity of microorganisms during storage, which has been reported to influence the optical properties of fermented dairy products [62].

**Figure 9:**
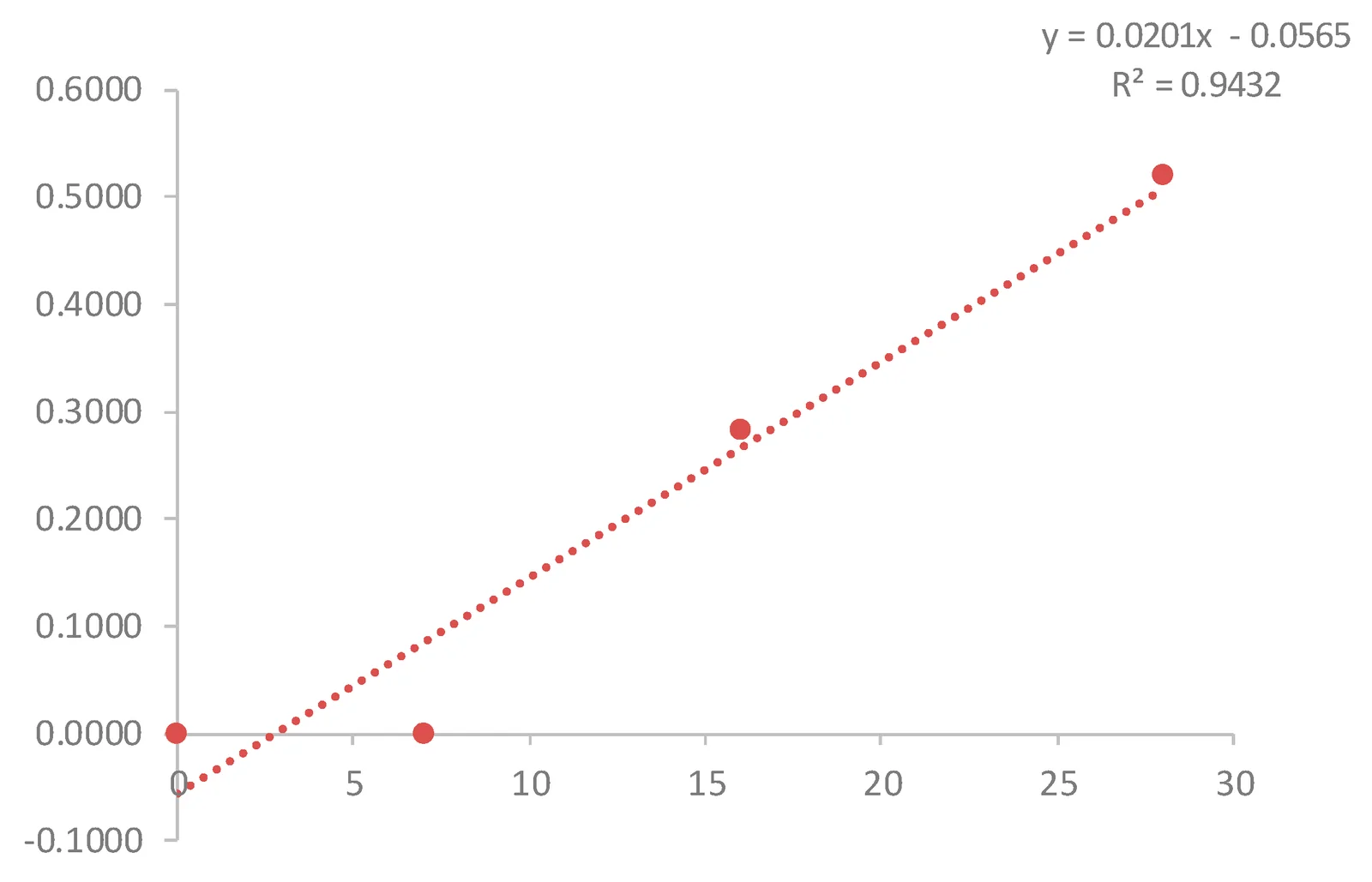
First-order stability kinetics of total phenolic compounds in the fermented dairy beverage during refrigerated storage. The graph represents the relationship between ln(C_0_/C_t_) and storage time (28 days). The slope of the linear regression corresponds to the degradation rate constant (k), while the half-life (t_1_/_2_) was calculated according to the first-order kinetic equation.

Regarding the stability kinetics, the data showed a good fit to a first-order kinetic model (R^2^ = 0.9432), with a degradation rate constant of 0.0201 day^−1^. The first-order model was selected based on its good fit to the experimental data (Figure 9).

Based on the estimated degradation rate constant, the half-life was calculated as 34.5 days. This value indicates that, according to the model, the time required for a 50% reduction in phenolic compound concentration would exceed the 28-day storage period evaluated in this study. The half-life represents a model-based estimate, obtained from four sampling points (T0, T7, T16 and T28), and extrapolates the period of experimental observation. Therefore, the kinetic analysis indicates considerable stability of the phenolic compounds during the evaluated 28-day refrigerated storage period. This behavior is consistent with studies reporting the maintenance of phenolic compounds in fermented dairy products during refrigerated storage, although stability may vary according to the matrix and the nature of the compounds [63,64].

Overall, these results demonstrate that the incorporation of microencapsulated HS phenolic compounds enabled the development of a fermented dairy beverage with satisfactory physicochemical stability, preservation of phenolic compounds and antioxidant activity, and acceptable color stability during refrigerated storage. Therefore, microencapsulation proved to be an effective strategy for incorporating natural bioactive compounds into dairy products, supporting the use of HS as both a functional ingredient and a natural colorant while contributing to the valorization of buttermilk as a sustainable dairy coproduct.

### 3.7. Phenolic Compound Profile in HS Calyces, HS Microcapsules, and Fermented Dairy Beverage

The phenolic compound profile of HS calyces, HS microcapsules, and the fermented dairy beverage was characterized by UPLC-MS/MS, with the identification and quantification of 14 compounds (Table 7). The identified compounds included mainly phenolic acids, flavonoids, and other phenolic compounds, in addition to methylxanthines, such as caffeine and theobromine. Among the detected compounds, isoferulic acid, caffeine, vanillic acid, p-coumaric acid, and 4-hydroxy-3-methoxycinnamaldehyde stood out, showing the highest concentrations in the analyzed samples. It is noteworthy that differences in the composition and concentrations of compounds among studies are expected, since the phenolic profile can be influenced by factors such as variety, geographical origin, cultivation conditions, processing, storage, extraction conditions, and the analytical method employed, among others [65,66,67,68].

The detection of the compounds identified in the HS extract also in the microcapsules and in the developed beverage indicates the maintenance of the phenolic profile of the extract after the microencapsulation process and its incorporation into the dairy matrix. This result is relevant from a technological perspective, as it demonstrates that microencapsulation enabled the incorporation of HS phenolic compounds into the final product. The literature highlights that the food matrix and processing conditions can modify the stability and availability of phenolic compounds, mainly due to interactions with other matrix componentes [69,70,71,72]. However, the results of the present study indicate that the conditions employed during microencapsulation and beverage formulation were adequate to preserve these constituents, minimizing potential losses during processing.

## 4. Conclusions

The present study demonstrated the feasibility of obtaining a phenolic-rich extract from HS and incorporating it into a fermented dairy beverage through microencapsulation. Among the extraction conditions evaluated, 70% cereal alcohol showed the highest extraction efficiency of phenolic compounds, flavonoids, and anthocyanins, as well as greater antioxidant capacity, and was therefore selected as the most suitable extraction condition. Among the encapsulation systems, the combination of 30% inulin and 70% maltodextrin obtained by spray drying showed the most favorable technological performance, combining an encapsulation efficiency of 69.98%, solubility above 90%, and predominantly spherical and structurally homogeneous particles. These characteristics supported its selection for application in the fermented dairy beverage.

The incorporation of the selected microcapsules resulted in a beverage with satisfactory physicochemical stability during 28 days of refrigerated storage and maintenance of the phenolic compounds and antioxidant capacity determined by ABTS. UPLC-MS/MS analysis confirmed the presence of 14 compounds in the *Hibiscus sabdariffa* calyces, microcapsules, and beverage, demonstrating the maintenance of these constituents during processing and their incorporation into the dairy matrix. Although changes in color and antioxidant capacity determined by FRAP were observed at the end of storage, the first-order kinetic model indicated relatively slow degradation of the phenolic compounds, with an estimated half-life of 34.5 days. Overall, the results demonstrate that HS microcapsules produced by spray drying with inulin and maltodextrin constitute an efficient strategy for preserving and incorporating phenolic compounds into a fermented dairy matrix, highlighting the potential of HS as a functional ingredient and natural colorant and contributing to the valorization of buttermilk as a dairy industry coproduct.

## 5. Patents

A patent application related to the fermented dairy beverage developed in this study is currently being filed through the Innovation and Technology Transfer Center (NIT) of the Federal University of Viçosa (UFV), Brazil.

## Figures and Tables

**Table 1:** Parameters used for the extraction of bioactive compounds from *Hibiscus sabdariffa* L.

Treatment	Solvent	Solvent Concentration
Extract 1	Ethanol	50% (*v*/*v*)
Extract 2	Ethanol	70% (*v*/*v*)
Extract 3	Ethanol	80% (*v*/*v*)
Extract 4	Cereal Alcohol	50% (*v*/*v*)
Extract 5	Cereal Alcohol	70% (*v*/*v*)
Extract 6	Cereal Alcohol	80% (*v*/*v*)

**Table 2:** Operational parameters used for the microencapsulation of *Hibiscus sabdariffa* L. phenolic compounds using different drying methods.

Treatment (Drying Method)	Encapsulating Agent (Concentration)
FD1	100% IN
FD2	30% IN:70% WPC
FD3	30% IN:70% MD
FD4	65% IN:35% WPC
FD5	65% IN:35% MD
FD6	30% IN:35% MD:35% WPC
SD1	100% IN
SD2	30% IN:70% WPC
SD3	30% IN:70% MD
SD4	65% IN:35% WPC
SD5	65% IN:35% MD
SD6	30% IN:35% MD:35% WPC

FD (Freeze-drying); SD (spray dryer); IN (inulin); WPC (whey protein concentrate); MD (maltodextrin); HS (*Hibiscus sabdariffa*).

**Table 3:** Concentration of total phenolic compounds, total flavonoids, total anthocyanins, and antioxidant capacity of concentrated extracts of *Hibiscus sabdariffa*.

Extracts	Total Phenolic Compounds	Total Flavonoids	Total Anthocyanins	ABTS	DPPH
	mg GAE/mL	mg CE/mL	mg C3GE/mL	mmol TE/mL	
E1	148.95 ± 5.45 ^c^	2.67 ± 0.56 ^ab^	0.57 ± 0.03 ^b^	909.52 ± 17.46 ^c^	5210.14 ± 18.12 ^c^
E2	170.74 ± 2.89 ^b^	2.59 ± 0.26 ^ab^	0.63 ± 0.09 ^b^	1023.81 ± 15.08 ^b^	6007.25 ± 18.12 ^c^
E3	83.09 ± 2.29 ^e^	1.73 ± 0.07 ^b^	0.32 ± 0.03 ^c^	723.81 ± 15.08 ^d^	1949.28 ± 19.98 ^d^
E4	129.45 ± 3.08 ^d^	1.83 ± 0.07 ^b^	0.79 ± 0.02 ^ab^	1041.07 ± 8.93 ^b^	4775.36 ± 19.28 ^c^
E5	208.04 ± 8.50 ^a^	3.04 ± 0.16 ^a^	1.37 ± 0.21 ^a^	1260.71 ± 19.05 ^a^	9539.85 ± 18.16 ^a^
E6	203.12 ± 5.39 ^a^	3.00 ± 0.04 ^a^	1.12 ± 0.08 ^a^	1205.36 ± 12.50 ^a^	7384.06 ± 18.12 ^b^

E1 (50% ethanol); E2 (70% ethanol); E3 (80% ethanol); E4 (50% cereal alcohol); E5 (70% cereal alcohol); E6 (80% cereal alcohol); GAE (gallic acid equivalents); CE (catechin equivalents); C3GE (cyanidin-3-glucoside equivalent); TE (Trolox equivalents). Different letters in the same column indicate a statistically significant difference (ANOVA and Tukey test, *p* < 0.05).

**Table 4:** pH values of HS phenolic compound microcapsules obtained by freeze-drying (FD) or spray drying (SD) using different encapsulating agents (inulin, maltodextrin, and concentrated whey protein).

Samples	pH
FD1	2.3 ± 0.02 ^a^
FD2	3.2 ± 0.10 ^a^
FD3	2.3 ± 0.02 ^a^
FD4	2.7 ± 0.05 ^a^
FD5	2.3 ± 0.02 ^a^
FD6	2.7± 0.04 ^a^
SD1	1.9 ± 0.08 ^a^
SD2	2.8 ± 0.07 ^a^
SD3	1.9 ± 0.08 ^a^
SD4	2.3 ± 0.02 ^a^
SD5	1.9 ± 0.08 ^a^
SD6	2.3 ± 0.02 ^a^

FD (Freeze-drying); SD (spray dryer). Different lowercase letters in the same column indicate a statistically significant difference (ANOVA and Tukey’s test, *p* < 0.05).

**Table 5:** Quantification of total phenolic compounds, total flavonoids, total anthocyanins, and antioxidant capacity of HS extract microcapsules.

Samples	Total Phenolic Compounds	Total Flavonoids	Total Anthocyanins	ABTS	DPPH
	(mg GAE/g)	(mg CA/g)	(mg C3G/g)	(mmol TE/g)	
FD1	284.44 ± 10.28 ^ab,A^	59.56 ± 2.43 ^a,A^	2.25 ± 0.03 ^b,A^	588.00 ± 8.82 ^a,A^	805.51 ± 10.04 ^c,A^
FD2	283.33 ± 13.57 ^ab,A^	68.78 ± 3.47 ^a,A^	3.94 ± 0.19 ^a,A^	641.89 ± 5.36 ^ab,A^	879.90 ± 59.56 ^ab,A^
FD3	337.78 ± 12.85 ^a,A^	35.67 ± 2.03 ^b,A^	3.52 ± 0.07 ^a,A^	631.33 ± 2.89 ^a,A^	706.96 ± 24.76 ^e,A^
FD4	306.67 ± 17.71 ^b,A^	69.33 ± 1.53 ^a,A^	1.91 ± 0.05 ^c,B^	651.89 ± 3.85 ^a,A^	950.43 ± 30.12 ^a,A^
FD5	256.30 ± 12.60 ^ab,A^	66.78 ± 3.56 ^a,A^	4.16 ± 0.06 ^a,A^	636.89 ± 5.36 ^a,A^	715.65 ± 18.10 ^d,A^
FD6	316.67 ± 17.28 ^b,A^	71.78 ± 5.19 ^a,A^	3.65 ± 0.02 ^a,A^	674.67 ± 11.55 ^a,A^	839.32 ± 30.16 ^c,A^
SD1	383.33 ± 14.14 ^a,A^	46.89 ± 4.62 ^b,A^	2.18 ± 0.02 ^c,A^	592.44 ± 8.22 ^b,A^	805.51 ± 12.63 ^a,A^
SD2	203.33 ± 14.14 ^c,A^	76.89 ± 6.71 ^a,A^	3.45 ± 0.11 ^a,A^	566.89 ± 8.22 ^c,B^	794.88 ± 19.30 ^a,B^
SD3	291.19 ± 12.41 ^a,B^	32.22 ± 2.36 ^c,A^	1.87 ± 0.02 ^c,B^	608.56 ± 3.47 ^a,A^	700.19 ± 13.07 ^b,A^
SD4	342.96 ± 11.85 ^b,A^	71.89 ± 3.02 ^a,A^	2.41 ± 0.30 ^b,A^	619.11 ± 18.36 ^a,B^	735.94 ± 14.49 ^b,B^
SD5	362.22 ± 12.57 ^ac,A^	46.11 ± 3.56 ^b,B^	3.83 ± 0.16 ^a,B^	597.44 ± 8.39 ^b,B^	702.13 ± 15.96 ^b,A^
SD6	213.33 ± 12.27 ^c,B^	70.22 ± 6.29 ^a,A^	3.73 ± 0.02 ^a,A^	615.22 ± 14.56 ^a,B^	729.18 ± 9.31 ^b,B^

FD (Freeze-drying); SD (spray dryer); GAE (gallic acid equivalents); CE (catechin equivalents); C3G (cyanidin-3-glucoside); TE (Trolox equivalents). Lowercase letters indicate significant differences between formulations within the same drying method. Different uppercase letters indicate significant differences between drying methods for corresponding formulations. Statistical analysis was performed using two-way ANOVA followed by Tukey’s multiple comparisons test (*p* < 0.05).

**Table 6:** Color parameters L*, a*, b*, C*, and h° of buttermilk-based fermented milk beverage samples incorporated with microencapsulated phenolic extract from *Hibiscus sabdariffa* L. during 28 days of storage at 8 °C.

Parameters	Storage Period			
	0	7	16	28
L*	53.33 ± 0.00 ^a^	53.09 ± 0.00 ^b^	54.95 ± 0.00 ^b^	46.90 ± 0.00 ^b^
a*	14.12 ± 0.01 ^a^	11.58 ± 0.0 ^b^	10.52 ± 0.0 ^b^	6.75 ± 0.01 ^b^
b*	0.53 ± 0.00 ^a^	2.24 ± 0.0 ^b^	2.65 ± 0.0 ^b^	3.33 ± 0.03 ^b^
C*	14.13 ± 0.01 ^a^	11.79 ± 0.00 ^b^	11.04 ± 0.00 ^b^	7.25 ± 0.01 ^b^
hº	2.17 ± 0.02 ^a^	10.98 ± 0.06 ^b^	17.57 ± 0.16 ^b^	21.44 ± 0.11 ^b^
ΔE	-	3.07 ± 0.02 ^a^	4.83 ± 0.03 ^b^	10.00 ± 0.00 ^c^

Data are presented as mean ± standard deviation of three technical replicates. Different lowercase letters within the same row indicate significant differences (*p* < 0.05) between each storage time and the initial time (day 0), according to ANOVA followed by Dunnett’s multiple comparisons test. For ΔE, different lowercase letters within the same row indicate significant differences between storage times according to ANOVA followed by Tukey’s multiple comparisons test.

**Table 7:** Phenolic compounds identified and quantified by UPLC-MS/MS in HS calyces, HS microcapsules, and fermented dairy beverage.

Phenolic Compound	HS Calyces (µg/g)	HS Microcapsules (µg/g)	Fermented Dairy Beverage (µg/g)
4-Hydroxybenzoic acid	0.017	0.016	0.016
4-Hydroxy-3-methoxycinnamaldehyde	0.041	0.041	0.041
Caffeine-1	0.057	0.057	0.057
Chalcone	0.042	0.042	0.042
Coniferyl alcohol	0.025	0.025	0.024
Ferulic acid	0.032	0.031	0.031
Isoferulic acid	0.091	0.097	0.091
Naringenin	0.011	0.011	0.011
p-Coumaric acid	0.047	0.047	0.047
Syringic acid	0.037	0.035	0.034
Theobromine	0.016	0.016	0.015
Vanillic acid	0.056	0.057	0.056
Vanillin	0.038	0.038	0.038

Results are expressed as µg/g of sample. HS: *Hibiscus sabdariffa*.

## Data Availability

The data presented in this study are available from the corresponding author upon reasonable request due to intellectual property restrictions.

## Abbreviations

ABTS	2,2′-Azino-bis(3-ethylbenzothiazoline-6-sulfonic acid)
ANOVA	Analysis of variance
AOAC	Association of Official Analytical Chemists
APHA	American Public Health Association
C3GE	Cyanidin-3-glucoside equivalents
CE	Catechin equivalents
CFU	Colony-forming units
DPPH	2,2-Diphenyl-1-picrylhydrazyl
EE	Encapsulation efficiency
ESI	Electrospray ionization
FD	Freeze-drying
FRAP	Ferric reducing antioxidant power
GAE	Gallic acid equivalents
HS	*Hibiscus sabdariffa* L.
IN	Inulin
INMET	Instituto Nacional de Meteorologia
LAB	Lactic acid bacteria
MD	Maltodextrin
MPC	Mass of phenolic compounds recovered in the microcapsules
MRM	Multiple reaction monitoring
MRS	de Man, Rogosa and Sharpe
NIT	Núcleo de Inovação e Tecnologia
PCA	Plate Count Agar
PVDF	Polyvinylidene fluoride
SD	Spray drying
SEM	Scanning electron microscopy
TBC	Total bacterial count
TE	Trolox equivalents
TPTZ	2,4,6-Tris(2-pyridyl)-s-triazine
UPLC-MS/MS	Ultra-high-performance liquid chromatography–tandem mass spectrometry
UFV	Universidade Federal de Viçosa
WPC	Whey protein concentrate
WSI	Water solubility index
